# Phonological Fluency Strategy of Switching Differentiates Relapsing-Remitting and Secondary Progressive Multiple Sclerosis Patients

**DOI:** 10.1155/2013/451429

**Published:** 2013-01-17

**Authors:** L. Messinis, M. H. Kosmidis, C. Vlahou, A. C. Malegiannaki, G. Gatzounis, N. Dimisianos, A. Karra, G. Kiosseoglou, P. Gourzis, P. Papathanasopoulos

**Affiliations:** ^1^Neuropsychology Section, Department of Neurology, University of Patras Medical School, 265 04 Patras, Greece; ^2^Laboratory of Cognitive Neuroscience, School of Psychology, Aristotle University of Thessaloniki, 54124 Thessaloniki, Greece; ^3^Department of Neurosurgery, University of Patras Medical School, 265 04 Patras, Greece; ^4^Department of Psychiatry, University of Patras Medical School, 265 04 Patras, Greece

## Abstract

The strategies used to perform a verbal fluency task appear to be reflective of cognitive abilities necessary for successful daily functioning. In the present study, we explored potential differences in verbal fluency strategies (switching and clustering) used to maximize word production by patients with relapsing-remitting multiple sclerosis (RRMS) versus patients with secondary progressive multiple sclerosis (SPMS). We further assessed impairment rates and potential differences in the sensitivity and specificity of phonological versus semantic verbal fluency tasks in discriminating between those with a diagnosis of MS and healthy adults. We found that the overall rate of impaired verbal fluency in our MS sample was consistent with that in other studies. However, we found no differences between types of MS (SPMS, RRMS), on semantic or phonological fluency word production, or the strategies used to maximize semantic fluency. In contrast, we found that the number of switches differed significantly in the phonological fluency task between the SPMS and RRMS subtypes. The clinical utility of semantic versus phonological fluency in discriminating MS patients from healthy controls did not indicate any significant differences. Further, the strategies used to maximize performance did not differentiate MS subgroups or MS patients from healthy controls.

## 1. Introduction

Multiple sclerosis (MS) is a chronic, debilitating, autoimmune disease of the nervous system that usually presents with a relapsing-remitting, and then later a progressive, course. Both the course of the illness and the presentation of motor and cognitive symptoms in terms of type and severity can vary significantly from one individual to another [1]. The disease has been classified as a frontal-subcortical dementia, as it causes demyelination of neurons mainly in frontal and subcortical regions [2].

It has been estimated that approximately 60% of patients with multiple sclerosis present with cognitive deficits [3]. Consistent with the locations of disease-induced lesions, subsequent functional impairments comprise problems with attention, information processing speed, memory, and executive functioning, all of which limit the individual's ability to perform within the context of work and social relationships and may even compromise the safety of daily activities such as driving [4].

Although the cognitive effects of MS have been repeatedly documented in previous studies [3], it is not clear whether cognitive deficits are exclusively the result of neurological damage or could also be the product of secondary symptoms of MS, such as depression and fatigue [5]. Further, individuals presenting with secondary progressive MS (SPMS) seem to consistently present with lower performance than those with relapsing-remitting MS (RRMS), possibly due to their older age, longer duration of illness, or more severe physical disability [3, 6, 7].

The strategies used to perform a verbal fluency test appear to be reflective of cognitive abilities necessary for successful daily functioning. Several studies have documented the association between verbal fluency performance and community functioning in patients with dementia [8] and those with schizophrenia [9]. Although community functioning was beyond the scope of the present study, we considered verbal fluency performance a potentially important indicator of cognitive processes used in situations requiring generation of an organized approach to achieve successful responding. In fact, performance of patients with MS on verbal fluency has been consistently recorded to be lower than in healthy controls. Henry and Beatty [10] conducted a review of 35 studies examining verbal fluency performance in MS. They found that patients with MS were substantially impaired on this measure, and that they presented with equal impairment on semantic and phonemic verbal fluency. The authors suggested that verbal fluency is one of the most sensitive measures of cognitive impairment in MS, along with the Symbol Digit Modalities Test of psychomotor speed. They also concluded that patients with SPMS presented with more severe deficits in comparison to patients with RRMS. In another study examining the use of strategies for maximizing word production, Tröster et al. [11] found that patients with MS produced more words than healthy controls; more interesting, however, was their compromised ability to switch between semantic or phonemic subcategories, despite producing an average number of words within each subcategory.

The aim of the current study was to explore (a) potential differences in strategies (switching and clustering) used to maximize word production by patients with RRMS versus SPMS, (b) impairment rates of verbal fluency (semantic and phonological) in MS patients, and (c) potential differences in the sensitivity and specificity of phonological versus semantic verbal fluency tasks in discriminating between those with a diagnosis of MS and healthy adults.

## 2. Method

### 2.1. Participants

Participants were 148 (91 females or 61.50%) native Greek-speaking individuals, recruited from southwestern and from northern Greece, who took part in the present study voluntarily, after providing a written informed consent for their participation. Seventy four of these individuals were diagnosed with clinically definite MS using McDonald's criteria [12]. Specifically, 60 (39 females or 65.0%) MS patients were diagnosed with RRMS (age: *M* = 41.18, SD = 11.08; level of education: *M* = 12.22, SD = 3.39, years) and 14 (10 females or 71.0%) were diagnosed with SPMS (age: *M* = 42.86, SD = 7.38; level of education: *M* = 12.07, SD = 3.29, years). MS groups differed from each other on the basis of physical disability status (*t*(62) = −2.920, *P* < 0.001), as determined by the Expanded Disability Status Scale (EDSS) [13] favouring the RRMS group (*M* = 3.22, SD = 0.914), which had less severe physical disability relative to the SPMS group (*M* = 6.14, SD = 0.949). The two patient groups also differed with respect to duration of illness (*t*(72) = −0.883, *P* < 0.001), with the RRMS group having slightly more than half the illness duration (*M* = 8.22, SD = 5.70 years) of the SPMS group (*M* = 14.86, SD = 6.03 years). The two groups did not differ on level of depression (*t*(72) = −2.150, *P* = 0.965), as assessed by the Beck Depression Inventory Fast Screen (BDI-FS) [14]. Both groups scored in the minimal depression range (RRMS group: *M* = 3.60, SD = 0.91; SPMS group: *M* = 4.10, SD = 1.04). All patients were receiving standard MS medications (immunomodulators) and symptomatic medication for reduction of spasticity, fatigue, and spasm, as prescribed by their attending neurologists with doses adjusted for optimal clinical benefit. We excluded participants from this MS group who suffered from any other medical condition (i.e., major psychiatric disorders, other neurological disorders, type II diabetes, traumatic brain injury, loss of consciousness >5 minutes, and hearing impairment not sufficiently corrected by a hearing aid) that might affect neuropsychological performance and nonnative speakers of the Greek language.

The remaining 74 (42 females or 56.75%) individuals were healthy adult participants (age: *M* = 42.45, SD = 11.26; level of education: *M* = 12.46, SD = 3.21, years) invited to take part in the study by their neurologist or family doctors, in order to improve study participation and compliance, or by a neuropsychologist. All healthy participants were screened with a medical questionnaire and physical examination for conditions that might influence cognitive performance. Exclusion criteria for the healthy participants were a history of psychiatric, neurological, or cardiovascular disorders or of substance abuse or dependence (including alcohol and benzodiazepine abuse), any other medical condition (including hearing impairment not sufficiently corrected by a hearing aid) that might affect neuropsychological performance, and nonnative speakers of the Greek language. We further excluded from the study potential participants who on initial testing obtained scores of less than 27 on the Greek validated version of the Mini Mental State Examination [15], a brief screening measure of global cognitive deficits.

### 2.2. Procedure

Healthy participants and MS patients were tested individually by psychologists in the clinic. Healthy participants were initially screened through a standardized interview at the beginning of the testing session by the project staff clinical neuropsychologist and physician, in order to exclude those with health problems or other exclusion criteria as described above. Healthy participants were also administered the Greek version of the Mini Mental State Examination [15]. The psychologists who tested the participants had been intensively trained in the administration procedures of various neuropsychological measures by doctoral-level clinical neuropsychologists.

All participants were assessed with the Greek Verbal Fluency Test [16]. The administration and scoring procedures were those proposed by Kosmidis et al. [16] and are described here briefly. On the semantic part of the test (categories), we asked participants to generate as many different animals, fruits, and objects as possible, each in a time period of 60 seconds. On the phonological part of the test (letters), we asked participants to generate as many different words as possible beginning with the Greek letters “*χ*” (chi), “*σ*” (sigma), and “*α*” (alpha), each in a time period of 60 seconds, excluding proper nouns and variations of the same word. Variables in the present analyses were the total number of words produced on the semantic task and the total number of words produced on the phonological task. We also analyzed the strategies utilized to maximize word generation: semantic and phonemic clustering (i.e., the process of organizing words into semantically or phonemically related subcategories) and switching (i.e., shifting between subcategories or clusters). Detailed scoring rules for switching and clustering are provided in the Appendix of this paper and in accordance with Kosmidis et al. [16].

### 2.3. Statistical Analysis

The normality assumption or homogeneity of variance of our data was initially confirmed for each variable using the *Kolmogorov-Smirnov* test. Total word production, number of words related by clusters, and the number of switches were analyzed with multivariate analysis of variance. Equality of means between the MS groups and the healthy group, were analyzed using independent sample *t*-tests. In cases where statistically significant differences were found between the variances of groups, the *t*-test of unequal variances was used and the degrees of freedom were estimated using the Welch-Satterthwaite approximation. Levene's test was employed in order to investigate the equality of variances. The level of statistical significance was set at *a* = 0.05. We also calculated the number of MS patients impaired on total number of words produced on the semantic and phonological task separately (using as impairment criterion scores of 1.5 and of 2 SD below age and education-corrected normative Greek data) [16]. We further conducted a Receiver Operating Characteristic (ROC) analysis to investigate whether the phonological fluency task was more sensitive in detecting those with a diagnosis of MS from the healthy group as compared with the semantic fluency task. All analyses were conducted using the SPSS 17.0 software.

## 3. Results

The two groups, that is, MS group (RRMS and SPMS patients as a single group) and controls did not differ significantly on age (*t*(146) = −3.570, *P* = 0.669) or level of education (*t*(146) = −1.337, *P* = 0.829). Further, the two MS groups (RRMS and SPMS) did not differ significantly from each other in age (*t*(72) = −2.152, *P* = 0.935) or level of education (*t*(72) = 0.145, *P* = 0.885). However, there were significantly more female participants in both groups (RRMS and SPMS patients as a single group and healthy controls), (*χ*_(1)_^2^ = 7.811, *P* < 0.001).

Total word production, number of words related by clusters, and number of switches produced by multiple sclerosis patients as a single group, but also RRMS and SPMS subtypes, and healthy controls were compared using an analysis of variance. We found significant main effects for semantic fluency total word production (*F*(2,145) = 10.269, *P* < 0.001), semantic fluency number of switches (*F*(2,145) = 5.106, *P* < 0.007), phonological fluency total word production (*F*(2,145) = 11.131, *P* < 0.001), phonological fluency number of words produced related by cluster) (*F*(2,145) = 15.851, *P* < 0.001), and phonological fluency number of switches (*F*(2,145) = 25.852, *P* < 0.001). Comparisons that were significant in the *post hoc* tests are presented in Table 1.

We also recorded the percentage of MS patients who scored in the impaired range on total number of words produced on the semantic and phonological task, using scores 1.5 and 2 SD below age and education-corrected normative Greek data (see Kosmidis et al. [16]) as an impairment criterion. Twenty-two (29.72%) of our patients had impaired performance on the semantic task and 25 patients (33.78%) had impaired performance on the phonological task, when the impairment criterion was set at 1.5 SD below age and education-corrected normative Greek data. These rates, however, decreased when the impairment criterion became more conservative and was set at 2 SD below age and education corrected normative Greek data. Specifically, 13 (17.56%) of our patients had impaired performance on the semantic task and 14 (18.98%) had impaired performance on the phonological task.

We further examined the contribution of possible moderator variables of verbal fluency performance on the total MS group, including EDSS (physical disability) score, duration of illness, and BDI-FS (depression severity) score using regression analyses. We found that duration of illness contributed significantly to phonological fluency number of switches (*B* = −0.639, SE = 0.262, *P* = 0.018). There were no other significant contributions of possible moderator variables.

**Figure 1:**
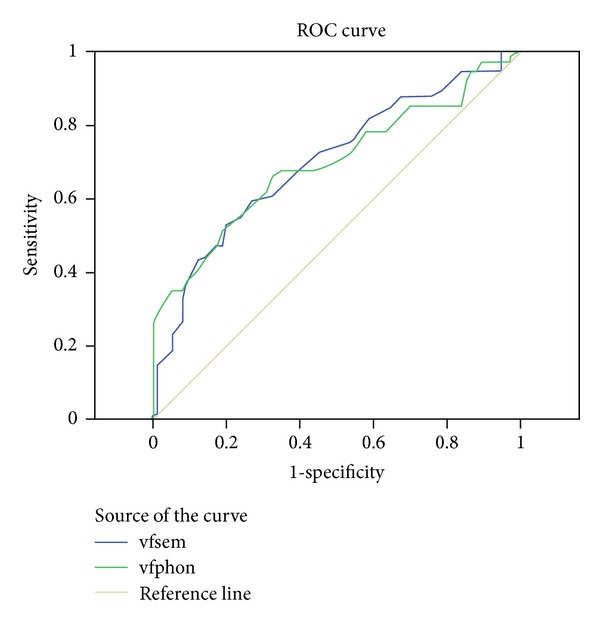
Diagonal segments are produced by ties. Region under the curve indicating similar sensitivity and specificity in both semantic and phonological word fluency.

**Figure 2:**
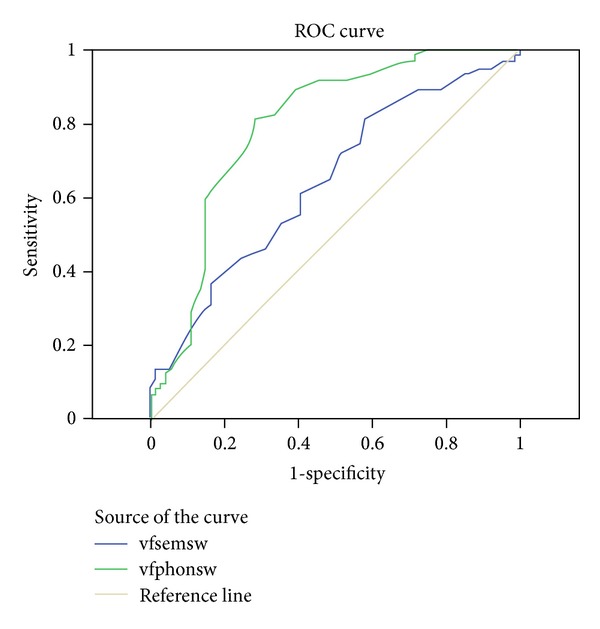
Diagonal segments are produced by ties. Region under the curve indicating similar sensitivity and specificity in both semantic switching and phonological switching in the word fluency task.

**Figure 3:**
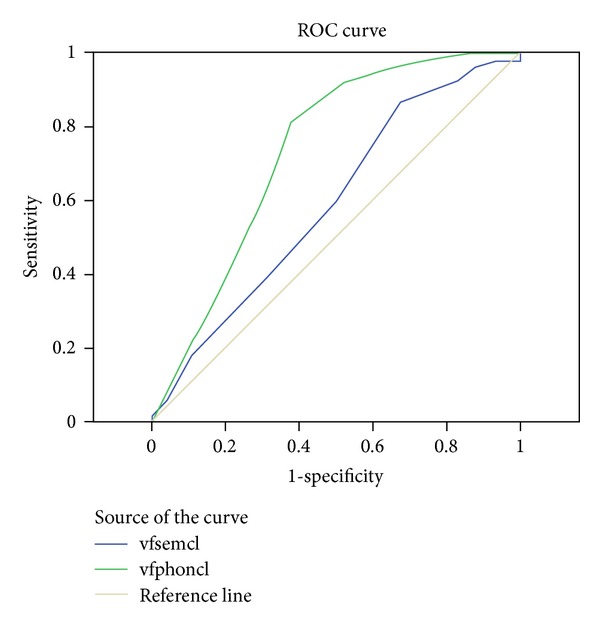
Diagonal segments are produced by ties. Region under the curve indicating similar sensitivity and specificity for semantic and phonological words related by clusters.

We also conducted a Receiver Operating Characteristic (ROC) analysis to investigate whether the phonological fluency test was more sensitive in detecting those with a diagnosis of MS from the healthy group as compared with the semantic fluency test. The area under the curve (Figure 1) was essentially identical for the total number of words produced on the two test conditions: 0.701 for the semantic fluency condition and 0.697 for the phonological condition. In both conditions, sensitivity and specificity were moderate and equal to each other. On the semantic condition, a score of 50.50 yielded 73% sensitivity and 54% specificity, with a positive predictive value of 61% and on the phonological condition a score of 34.50 yielded 72% sensitivity and 47% specificity, with a PPV of 58%. Similarly, the other variables of the word fluency test did not differentiate the conditions from each other with respect to their sensitivity and specificity. More specifically, the area under the curve (Figure 2) for semantic and phonological switches was 0.642 and 0.797, respectively. On the semantic condition, a score of 24.00 yielded 72% sensitivity and 49% specificity, with a PPV of 64% and on the phonological condition a score of 18.00 yielded 92% sensitivity and 55% specificity, with a PPV of 87%. Finally, the area under the curve (Figure 3) for semantic and phonological words related by clusters was 0.588 and 0.733, respectively. On the semantic condition, a score of 10.00 yielded 74% sensitivity and 40% specificity, with a PPV of 51% and on the phonological condition a score of 4.00 yielded 92% sensitivity and 47% specificity, with a PPV of 36%.

## 4. Discussion

The present study was conducted to assess verbal fluency functioning in multiple sclerosis (MS) patients, including strategies utilized to maximize word production, and the clinical utility of phonological versus semantic fluency in discriminating healthy adults from MS patients. We also calculated the overall prevalence of verbal fluency impairments in these patients.

We found that the overall rate of impaired verbal fluency in our MS sample was consistent with that in other studies [6, 10]. However, unlike the majority of previous studies, which have found better performance for RRMS patients compared to the progressive subtypes [10, 17, 18], we found no differences between types of MS (SPMS, RRMS), on semantic or phonological fluency word production, or the strategies (clusters and switches) used to maximize semantic fluency. Recently, Potagas et al. [6] did not find significant differences on a semantic word list generation task when comparing Greek SPMS patients to the other subtypes. This finding is consistent with our findings, particularly as related to the semantic component of the verbal fluency test that we used in this study.

In contrast, we found that the number of switches differed significantly in the phonological fluency task between the SPMS and RRMS subtypes. This finding appears to be related to the duration of illness, which is significantly longer for the SPMS subtype, as all other moderator variables (EDSS, depression status, age) that we examined did not contribute to total fluency production or the strategies utilized.

Further, the performance of the MS patients, either as a single group or as MS subtypes, differed significantly from the healthy adults, showing poorer performance in total word production for both conditions and for the strategies utilized to achieve maximum word production. The finding that our MS patients' performance differed significantly as compared to the healthy group is also consistent with previous studies [10, 17, 19].

Regarding the clinical utility of the verbal fluency condition (semantic versus phonological) in discriminating MS patients from healthy controls, we did not find any significant differences in sensitivity and specificity (according to the ROC curve analyses). Further, the strategies used to maximize performance (clustering and switching) did not differentiate MS subgroups or MS patients from healthy controls, as we again did not find any significant differences in sensitivity and specificity for these variables. Our finding is in contrast to a previous study [19] that found a semantic fluency measure to discriminate adequately between MS patients and controls. Furthermore, in a recent quantitative review article of 35 studies, the authors noted that phonemic and semantic fluency tests are equally sensitive to MS, therefore, encouraging the view that the results on fluency tests for different languages will be comparable. This finding however, requires further investigation, as our findings do not support this hypothesis, at least not for the Greek language. Several potential limitations to the generalizability of our findings should be mentioned. Firstly, the relatively small number of SPMS patients reduced the statistical power of detecting significant differences between the MS subtypes. Secondly, the inclusion of primary progressive multiple sclerosis (PPMS) patients and possibly patients with a clinically isolated syndrome (CIS) suggestive of MS would have provided more information and possibly revealed a pattern of verbal fluency performance in multiple sclerosis.

In conclusion, our findings do not support differences between the SPMS and RRMS subtypes, in verbal fluency performance, or the strategies utilized to achieve word production, with the exception of number of switches which differed significantly in the phonological fluency task. Further, semantic-versus-phonological fluency do not appear to adequately discriminate MS patients from healthy controls or SPMS from RRMS patients. In contrast, we found a rate of impaired verbal fluency consistent with previous studies.

## Figures and Tables

**Table 1:** Verbal fluency performance for healthy and multiple sclerosis groups—*M *(SD).

	Healthy group (*n* = 74)	MS group (*n* = 74)	RRMS subgroup (*n* = 60)	SPMS subgroup (*n* = 14)	*P *
Variable					
Semantic fluency					
Word production^1,2,3^	51.09 (9.44)	43.72 (10.57)	44.13 (10.23)	41.93 (12.16)	∗∗^1^ ∗∗^2^ ∗^3^
Related words (clusters)	9.38 (4.49)	9.93 (2.93)	9.83 (2.83)	9.36 (3.43)	
Switches^1,2,3^	29.46 (9.03)	25.27 (7.10)	25.57 (7.05)	24.00 (7.47)	∗^1^ ∗^2^ ∗^3^
Phonological fluency					
Word production^1,2,3^	35.53 (9.84)	27.54 (12.26)	28.60 (11.34)	23.00 (11.22)	∗∗^1^ ∗^2^ ∗^3^
Related words (clusters)^1,2,3^	2.24 (1.55)	4.39 (2.89)	4.45 (3.02)	4.14 (2.31)	∗∗^1^ ∗^2^ ∗^3^
Switches^1,2,3,4^	29.22 (8.60)	19.27 (9.57)	20.50 (9.78)	14.00 (6.59)	∗∗^1^ ∗∗^2^ ∗∗^3^ ∗^4^

MS: multiple sclerosis group (as a single RRMS and SPMS group); RRMS: relapsing-remitting multiple sclerosis subgroup; SPMS: secondary progressive multiple sclerosis subgroup; Healthy group: healthy control group.

*P < 0.05.

**P < 0.001.

Healthy versus MS (as a single RRMS and SPMS group)^1^; Healthy versus RRMS^2^; Healthy versus SPMS^3^; RRMS versus SPMS^4^.

## Appendix

### A. Scoring Rules for Clustering and Switching

#### A.1. Semantic Clusters and Switches

We considered three or more consecutive words belonging to the same semantic subcategory a semantic cluster. We calculated semantic switches (SW; number of transitions between clusters, including single words) by subtracting the total number of related words (RW; all words belonging to a semantic cluster) from the total word production (WP) and adding that to the number of semantic clusters (SC): (WP − RW) + SC = SW.

Two of the authors (C. H. Vlahon and M. H. Kosmidis) determined subcategory groups based on naturally occurring clusters in the participants' protocols and familiarity of Greek individuals with items. For example, most Greeks will be familiar with a variety of farm animals, while they may not be as familiar with animals of Africa or the Arctic/Far North. Accordingly, Greeks tend to group fruit based on the time of the year at which they are ripe. When two consecutive words with a strong association in the Greek language were mentioned, they, too, were considered a cluster. We created the following list as a guide in determining strong pairs of words, as well as semantic subcategories.

##### A.1.1. Animals

1. Strong pairs: cat-dog, bunny-rabbit, turtle-rabbit, cat-mouse, donkey-horse-mule (two out of three), mouse-rat, lion-tiger, hawk-eagle, fox-wolf, fox-chicken, wolf-lamb, and elephant-mouse.
2. Subcategories: they are as the following.
  - Farm animals: cow, ox, goat, lamb, billy goat, bull, chicken, rooster, dog, horse, donkey, mule, rabbit, duck, goose, and so forth.
  - Animals of the Greek forest: wolf, bear, fox, squirrel, raccoon, skunk, wild boar, porcupine, deer, weasel, beaver, badger, and so forth; birds of this category such as owl, eagle, hawk, cuckoo, and crow; and snakes of this category such as viper.
  - Tropical animals, animals of the steppe, animals of the jungle, and safari animals: crocodile, elephant, hippopotamus, rhinoceros, tiger, lion, puma, antelope, zebra, giraffe, buffalo, camel, kangaroo, koala, primates such as monkey and gorilla; birds of this category such as flamingo, parrot, and vulture; and snakes of this category such as cobra and python.
  - Reptiles: crocodile, all types of snakes, turtle, iguana, lizard, frog, chameleon, and so forth.
  - Birds.
  - Fish, including anything living underwater such as mammals (e.g., dolphin, whale) or shells.
  - Insects.

##### A.1.2. Fruit

1. Strong pairs: apple-orange, orange-tangerine, cherry-sour cherry, apple-pair, melon-watermelon.
2. Categories: they are as the following.
  - Winter fruit.
    - Citrus fruits: orange, tangerine, lemon, bitter orange, grapefruit.
    - Apple, pair, kiwi, quince, and so forth.
  - Spring and summer fruit.
    - Tropical fruit: avocado, mango, pineapple, coconut, banana, papaya, and so forth.
    - Peach, apricot, nectarine, cherry, strawberry, watermelon, fig, grape, berry, melon, pomegranate, cranberry, plum, and so forth.
  - Dry fruit: fig, plum, walnut, peanut, hazelnut, almond, and so forth.
  - Furniture.
  - Appliances.
  - Clothes.
  - Linens-rugs: curtain, pillowcase, bed-sheet, pillow, carpet, towel, doily, doormat, embroidery, tablecloth, and so forth.
  - Household items: door, window, doorknob, blinds, chimney, fireplace, staircase, toiletries, gate, doorbell, lock, radiator, shutters, and so forth.
  - Kitchen items: cookware, Tupperware, utensils, cup, glass, kitchen appliances (such as oven, refrigerator)
  - Office items: desk, chair, paper, computer, stationery, pen, pencil, eraser, notebook, and so forth.
  - Decorative items: vase, chandelier, painting, poster, crystal-ware, porcelains, religious icons, ashtray, lamp, and so forth.
  - Tools: hammer, nail, drill, screwdriver, saw, rake, hose, shovel, pincer, spade, pick, and so forth.
  - Vehicles.
  - Jewelry.
  - Cosmetics and accessories: perfume, shampoo, hair band, comb, watch, wallet, aftershave, razor, sponge, nail polish, and so forth.

##### A.1.3. Objects

1. Strong pairs: broom-dustpan, table-chair, cigarette-lighter-ashtray (two out of three), pencil-eraser, pencil-workbook, and hammer-nail.
2. Categories: they are as the following.
  - Furniture.
  - Appliances.
  - Clothes.
  - Linens-rugs: curtain, pillowcase, bed-sheet, pillow, carpet, towel, doily, doormat, embroidery, tablecloth, and so forth.
  - Household items: door, window, doorknob, blinds, chimney, fireplace, staircase, toiletries, gate, doorbell, lock, radiator, shutters, and so forth.
  - Kitchen items: cookware, Tupperware, utensils, cup, glass, kitchen appliances (such as oven, refrigerator)
  - Office items: desk, chair, paper, computer, stationery, pen, pencil, eraser, notebook, and so forth.
  - Decorative items: vase, chandelier, painting, poster, crystal-ware, porcelains, religious icons, ashtray, lamp, and so forth.
  - Tools: hammer, nail, drill, screwdriver, saw, rake, hose, shovel, pincer, spade, pick, and so forth.
  - Vehicles.
  - Jewelry.
  - Cosmetics and accessories: perfume, shampoo, hair band, comb, watch, wallet, aftershave, razor, sponge, nail polish, and so forth.

#### A.2. Phonemic Clusters and Switches

We considered three or more consecutive words beginning with the same two letters and having the same sound (e.g., gallant-gap-gas), two consecutive words that differed only in a vowel sound (e.g., rule-role), or words that were homophones (e.g., route-root) as a phonemic cluster. We estimated phonemic switches (SW) by subtracting the total number of words related to each phonemic cluster (RW) from the total phonemic word production (WP) and adding that to the number of phonemic clusters (PC): (WP − RW) + PC = SW.

If two or more successive words stemmed from the same root (such as act-action-acting), we considered them repetitions and, thus, did not calculate a cluster based on them. If the words only shared a part/suffix but had a different meaning (e.g., superman-supermarket-supercilious), however, we considered them a cluster.
